# *Oreocharis tetrapterus* (Gesneriaceae), a new species from East Guangxi, China

**DOI:** 10.3897/phytokeys.131.35434

**Published:** 2019-09-13

**Authors:** Bo Pan, Guang-Da Tang, Truong Van Do, Stephen Maciejewski, Chong-Lang Deng, Fang Wen

**Affiliations:** 1 Guangxi Key Laboratory of Plant Conservation and Restoration Ecology in Karst Terrain, Guangxi Institute of Botany, Guangxi Zhuangzu Autonomous Region and Chinese Academy of Sciences, Guilin 541006, China Guangxi Institute of Botany, Guangxi Zhuangzu Autonomous Region and Chinese Academy of Sciences Guilin China; 2 Gesneriad Conservation Center of China (GCCC), Guilin Botanical Garden, Chinese Academy of Sciences, Guilin 541006, China Guilin Botanical Garden, Chinese Academy of Sciences Guilin China; 3 South China Limestone Plants Research Center, College of Forestry and Landscape Architecture, South China Agricultural University, Guangzhou 510642, China South China Agricultural University Guangzhou China; 4 Henry Fok College of Life Science, Shaoguan University, Shaoguan 512000, China Shaoguan University Shaoguan China; 5 Vietnam National Museum of Nature, Vietnam Academy of Science & Technology, 18 Hoang Quoc Viet, Hanoi, Vietnam Vietnam National Museum of Nature, Vietnam Academy of Science & Technology Hanoi Vietnam; 6 The Gesneriad Society, 2030 Fitzwater Street, Philadelphia, PA 19146, USA The Gesneriad Society Philadelphia United States of America; 7 Gupo Mountain Autonomous Region Nature Reserve, Guangxi, Hezhou 542899, China Gupo Mountain Autonomous Region Nature Reserve Hezhou China

**Keywords:** Didymocarpinae, Didymocarpoideae, Flora of Guangxi, Gupo Mountain Area, New taxon, Trichosporeae

## Abstract

A new species, *Oreocharis tetrapterus* F.Wen, B.Pan & T.V.Do (Gesneriaceae) from Gupo Mountain area, Hezhou city, Guangxi Zhuangzu Autonomous Region, China, is described and illustrated. The new species has a zygomorphic tetramerous corolla with two adaxial and two abaxial lobes and two fertile stamens in the posterior position, making this a unique combination of floral characteristics in the expanded *Oreocharis*.

## Introduction

In 2011, *Oreocharis* Bentham was redefined (Möller et al. 2011), and soon afterwards, this genus rapidly grew with the addition of many new species. *Oreocharissensu lato* now comprises at least 125 species (Möller et al. 2016, Möller 2019). *Oreocharis* is quasi-endemic to China because there are about 14 species distributed in other countries apart from China: namely, *O. primuloides* (Miq.) Benth. & Hook.f. ex Clarke (Japan), *O. hirsuta* Barnett (Thailand), *O. muscicola* (Craib) Mich.Möller & A.Weber (Bhutan, India, Myanmar), *O. longifolia* (Craib) Mich.Möller & A.Weber (Myanmar) and nine species from Vietnam (Wang et al. 1990, 1998, Li and Wang 2004, Wei et al. 2010, Do et al. 2017, Chen et al. 2017, 2018, Möller et al. 2018, Möller 2019).

A joint expedition from the Gesneriad Conservation Center of China (GCCC), Guilin Botanical Garden and Vietnam National Museum of Nature yielded collections of flowering specimens and living plants of an unidentified species of Gesneriaceae in August 2016. Plants raised in cultivation in the GCCC greenhouse from these collected living plants and seeds flowered in 2018. We carefully observed its habit (leaves in basal rosette), flower shape (infundibuliform corolla), number of fertile stamens (two, free, in the posterior position), filament shape (nearly straight) and capsule shape (long and cylindrical bivalved capsules with loculicidal dehiscenc), and identified it as belonging to the expanded *Oreocharis* (Wang et al. 1990, 1998; Möller et al. 2011). The expanded *Oreocharis* includes species of the former *Opithandra* B.L. Burtt (Burtt 1958) that were characterized by two stamens in the posterior position similar to the new species described here (Möller et al. 2011).

Following a careful review of the relevant herbarium specimens and taxonomic publications of *Oreocharis* from Guangxi and adjacent regions, we concluded that this species is new to science. The unusual characteristics of two stamens and zygomorphic flower with 2-lobed upper and lower lips are very rare in the expanded *Oreocharis*. *Oreocharis tetrapterus* F. Wen, B. Pan & T.V. Do is described and illustrated below.

## Material and methods

We performed and described the measurements and morphological character assessments of the new species by using collected specimens by BP, GDT, TVD, CLD and FW, living material observed in the field, and cultivated at the nursery of GCCC. All available *Oreocharis* specimens of China, Thailand and Vietnam, stored in the following herbaria were examined: E, GH, HN, IBK, K, KUN, MO, PE, PH, US and VNMN. At the same time, specimen images and name lists of the above-mentioned species (all species belong to former *Opithandra* but now transferred to *Oreocharis*) were obtained and checked from Tropicos (http://www.tropicos.org), JSTOR Global Plants (http://plants.jstor.org), The Plant List (http://www.plantlist.org/) and the International Plant Names Index (http://www. ipni.org). We studied all morphological characters under dissecting microscopes, and described the morphological identification and characters of this new species by using the terminology used by Wang et al. (1998).

## Taxonomic treatment

### Oreocharis tetrapterus

Taxon classificationPlantaePasseriformesParamythiidae

F.Wen, B.Pan & T.V.Do sp. nov.

4DD82562D4B95A4299EF2FEE924E730B

urn: lsid: ipni.org: names:60479368-2

**Figure 1.**
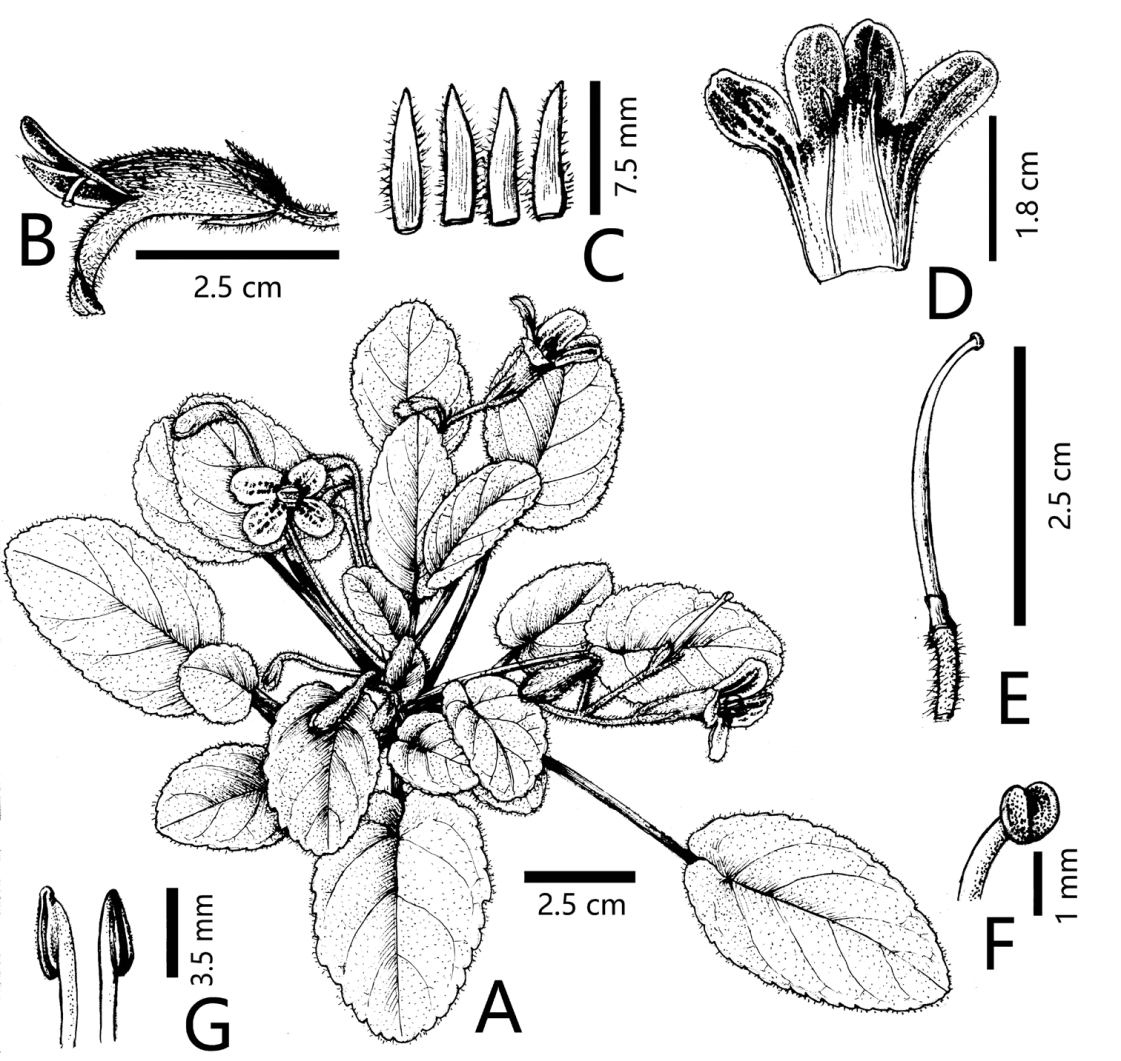
*Oreocharis tetrapterus* F.Wen, B.Pan & T.V.Do sp. nov. **A** habit **B** lateral view of flower **C** adaxial surfaces of calyx lobes **D** opened corolla for showing the two fertile stamens in posterior position **E** pistil with disc, sepals removed **F** stigma **G** anthers in side view. Drawn by Wen-Hong Lin from the holotype.

**Figure 2.**
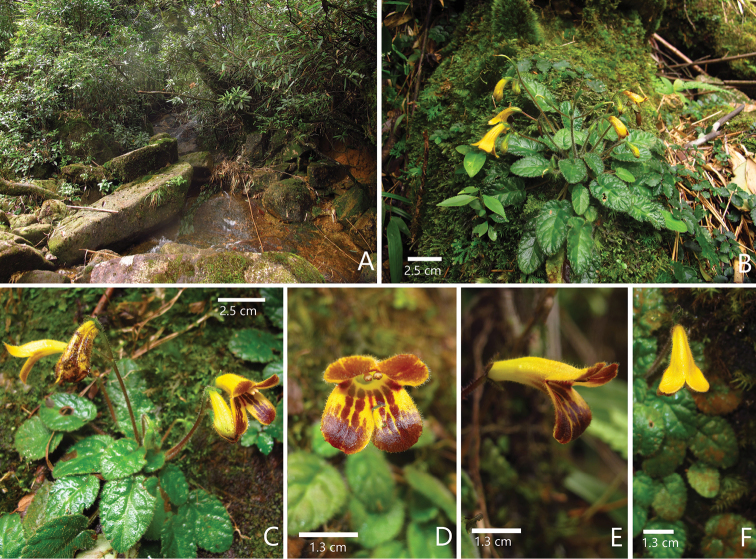
*Oreocharis tetrapterus* F.Wen, B.Pan & T.V.Do sp. nov. in its natural habitat **A** habitat **B** flowering plant **C** plant with flowering cymes **D** frontal view of corolla **E** lateral view of corolla **F** top view of corolla. Photographed by Bo Pan and Fang Wen, charted by Wen-Hua Xu.

**Figure 3.**
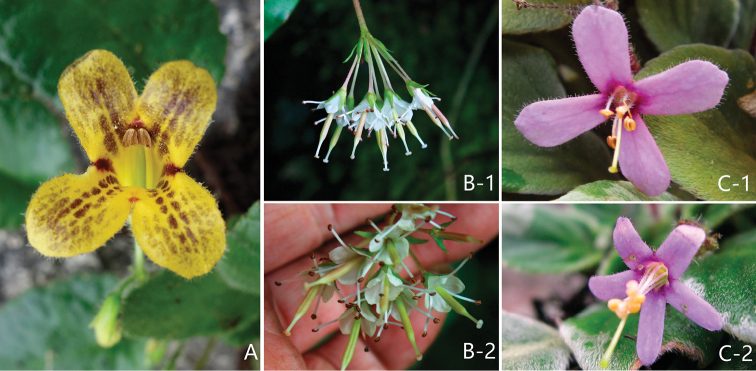
*Oreocharis tetrapterus* F.Wen, B.Pan & T.V.Do sp. nov. **A** lateral view of flower **B** top view of flower **C** lateral view of corolla and pistil and calyx lobes **D** adaxial surface of calyx lobes **E** abaxial surface of calyx lobes **F** pistil, sepals removed **G** opened corolla for showing stamens in posterior position **H** anthers, abaxial view? **I** adaxial leaf surface **J** abaxial leaf surface **K** peduncle indumentum. Photographed by Fang Wen and Bo Pan in the field, charted by Wen-Hua Xu.

**Figure 4.**
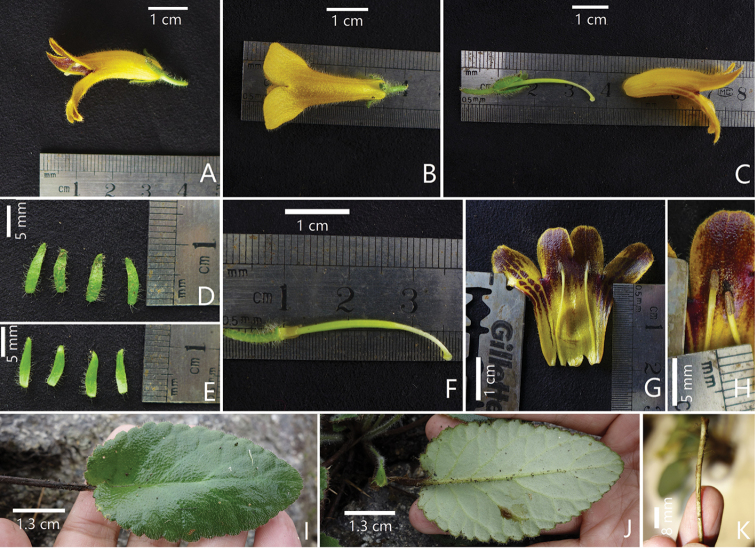
Some species in *Oreocharis* with typically four corolla lobes **A***Oreocharis tetrapterus* F.Wen, B.Pan & T.V.Do sp. nov. **B***O. sinensis* (Oliv.) Mich.Möller & A.Weber (**1** Lateral view of flowering cyme **2** Frontal view of flowering cyme) **D***O. esquirolii* Léveillé (**1** Corolla with four lobes and four stamens **2** Corolla with five lobes and five stamens). Photographed by Fang Wen and Bo Pan, charted by Wen-Hua Xu.

Figures 1 , 2 , 3 , 4A

#### Diagnosis.

The large bright yellow corolla is 2 lobed with the adaxial and abaxial lips both consistently 2-lobed, with irregular dark reddish-brown spots on the interior surfaces of the corolla lobes and 2 fertile stamens in posterior position distinguishes *Oreocharis tetrapterus* from all other species of *Oreocharis* s. l.

#### Type.

China. Guangxi: Hezhou City, Lisong Town, Gupo Mountain, 24°39'N, 111°36'E, elev. ca. 950 m, on moist surface of granite rocks, in flowering, 25 August 2018, *Wen Fang WF160825-01* (holotype: IBK!, isotype: IBK!).

#### Description.

Perennial herb, rhizome stem inconspicuous, 4–10 mm long, 3–4 mm in diam. Leaves 8–14, in basal rosette; petiole cylindric, 1–5 cm long, 2–3 mm in diam., sparsely to densely curly brown pubescent; leaf blade green to dark green, ovate to broadly elliptic, 3.0–5.5 × 2.2–3.5 cm, adaxially pubescent with nearly erect white hairs, abaxially sparsely pubescent to nearly glabrous but with dense white, slightly curly pubescence along main and lateral veins, lateral veins 5–6 on each side of midrib, adaxially inconspicuously sunk, adaxially conspicuously raised, apex obtuse to rounded, base often slightly asymmetric, margin crenate with 15–20 teeth on each side, more obvious on the lower half. Cymes axillary, inflorescence (1-)2–4-flowered; peduncle greenish brown to brown, 4–8 cm long, ca. 1.5 mm in diam., densely white pubescent; bracts 2, opposite, lanceolate to linear, ca. 5.0 × 1.0–1.5 mm, adaxially appressed white pubescent, abaxially nearly glabrous, margin nearly entire; pedicel green, 7–12 mm long, ca. 1 mm in diam., pubescent with dense, nearly erect hairs . Calyx green, 4-lobed to the base, lobes nearly equal, linear, 6–8 mm long, ca. 1.2 mm wide at base, outside white pubescent, inside glabrous. Corolla 2.2–2.8 cm long, bright yellow, inner side of corolla lobes with irregular dark reddish-brown spots, sometimes entire upper lobes reddish-brown, outside densely white glandular- and eglandular-pubescent, inside glandular puberulent in the throat and on adaxial lobes, tube broadly infundibuliform, 1.8–2.5 cm long, 6.5–8.5 mm in diam.; limb 2-lipped; adaxial lip 2-lobed divided to more than halfway, lobes broadly oblong to semiorbicular, 5–7 × 7–8 mm, abaxial lip 2-lobed to base, oblong, 8–10 × 5.5–7 mm. Stamens 2, in posterior position, 1.5–1.8 cm long, adnate to corolla 6–8 mm from base; filaments linear, yellow, glabrous; anthers narrowly horseshoe-shaped, apex acute, 2-loculed, dehiscing longitudinally; staminode 1, ca. 1.5 mm long, inserted at tube base. Disc tubular, ca. 5 mm high, yellowish green, margin undulate. Pistil 2.5–3 cm long when all corolla lobes outspread and flower completely opened; ovary green, cylindrical, glabrous, 1.8–2 cm long; style pale green, glabrous, 6–10 mm long; stigma bilobed, flabellate, pale green. Capsule linear, dehiscent but commonly one side of the capsule dehiscent first, loculicidal, straight in relation to pedicel, 3.5–4.5 cm long.

#### Phenology.

Flowering in August; fruiting in October.

#### Etymology.

The specific epithet, ‘tetrapterus’ from the Greek meaning having four wings or wing-like appendages. Here it refers to the four ‘wing-like’ lobes of the corolla, with adaxial and abaxial lips both having 2 lobes.,

#### Vernacular name.

The Chinese name of this new species is 姑婆山马铃苣苔. The pronunciation of the Chinese of this species is ‘Gū Pó Shān Mǎ Líng Jù Tái’.

#### Distribution and ecology.

*Oreocharis tetrapterus* is currently known only from one population of ca. 50 individuals at the type locality. The species may be endangered, but more data is needed to evaluate this reliably. The species grows on moist surfaces, on moss-covered granite rocks with other plants under subtropical bamboo and evergreen broad-leaved forest cover in Hezhou City, Guangxi.

#### Notes.

We understand most other Gesneriaceae with two stamens have them in the anterior position, but this special character, two stamens in the posterior position, has also evolved outside the *Oerocharis* s. l. in the South American *Sarmienta* Ruiz & Pavon (Ruiz and Pavon 1794) and the Asian/African *Epithema* Blume (Blume 1826, Bransgrove and Middleton 2015). *Oreocharis tetrapterus* is morphologically unique within *Oreocharis* s. l. and can be easily distinguished from the other species with four lobes by its zygomorphic corolla, whereas the others are actinomorphic, for example *O. sinensis* and *O. esquirolii* (*O. esquirolii* also has 5-lobed corolla type, occasionally) (Fig. 4).

## Supplementary Material

XML Treatment for Oreocharis tetrapterus
